# Effectiveness comparison between blended learning of histology practical in flipped physical classrooms and flipped virtual classrooms for MBBS students

**DOI:** 10.1186/s12909-022-03740-w

**Published:** 2022-11-16

**Authors:** Jinjie Zhong, Zhongjie Li, Xinyang Hu, Linlin Wang, Yingying Chen

**Affiliations:** 1 The Department of Basic Medical Science, and The Medical Education Research Center, School of Medicine, Zhejiang University, Yuhangtang Road 866, Xihu District, Hangzhou, 310058 China; 2 The Department of Basic Medical Science, School of Medicine, Zhejiang University, Hangzhou, 310058 China; 3 The Manchester Institute of Education, School of Environment, Education and Development, University of Manchester, Manchester, M13 9PL UK

**Keywords:** Blended learning, Flipped in physical classroom, Flipped in virtual classroom, Traditional learning, Student score, Satisfaction, Histology practical, Bachelor of medicine, bachelor of surgery (MBBS) student

## Abstract

**Background:**

The flipped classroom blended learning model has been proven effective in the teaching of undergraduate medical courses as shown by student acceptance and results. Since COVID-19 necessitated the application of online learning in Histology practical for MBBS students, the effectiveness of the blended learning model on teaching quality has required additional attention.

**Methods:**

A blended learning of histology practical was flipped in a virtual classroom (FVCR-BL) or in a physical classroom (FPCR-BL) in School of Medicine, Zhejiang University in China. Students were split into FVCR-BL group (*n* = 146) due to COVID-19 pandemic in 2020 or were randomly allocated into FPCR-BL group (*n* = 93) in 2021, and retrospectively, students with traditional learning in 2019 were allocated into traditional learning model in a physical classroom (PCR-TL) group (*n* = 89). Same learning requirements were given for 3 groups; all informative and summative scores of students were collected; a questionnaire of student satisfaction for blended learning activities were surveyed in 2021. Data of scores and scales were analyzed with Kruskal–Wallis test and Kolmogorov–Smirnov test in SPSS Statics software.

**Results:**

The results clarified that FPCR-BL students obtained higher final exam scores and were more likely to engage in face-to-face interactions with instructors than FVCR-BL students. FPCR-BL and FVCR-BL students had higher classroom quiz scores than the PCR-TL students owing to the contribution of blended learning. The results of the questionnaire showed that participants of FPCR-BL positively rated the online learning and preview test, with a cumulative percentage of 68.31%, were more satisfying than other learning activities of blended learning. There were significant correlations (*r* = 0.581, *P* < 0.05) between online learning and the other three blended learning strategies.

**Conclusions:**

In the flipped classroom with a blended learning process of histology practical, enhancing the quality of online learning boosts student satisfaction and improves knowledge learning; peer-to-peer interactions and instructor-to-peer interactions in the physical classroom improved knowledge construction.

## Background

In the histology practical, undergraduate students in medical school usually read the normal microstructures from glass or virtual slides and identify relationships between various cells and tissues of the human body to understand their corresponding functions. In a general medicine degree program in China, 32–36 classroom hours in the histology practical are instructed by teachers in late academic year 1 or year 2–3, depending on the curriculum.

In contrast to the USA and other countries, the MBBS students in China’s 5-year program who are enrolled from high school directly are required to complete 5.5–6.0 classroom hours in each class day for general arts courses and fundamental medical courses (such as systematic anatomy and histology) during year 1. This means that histology educators and learners must keep up with the relevance and depth of the comprehensive knowledge required [1, 2] and know how to apply histology to related courses, clinical cases, and medical research [3] in the current integrated curriculum trends [4, 5]. Therefore, new approaches of histology learning should fit the needs and workloads of MBBS students in China. Blended learning is one such method for facing the challenges of this unique era.

Blended learning is an educational strategy that combines traditional classroom activities with online activities in a flipped environment, where the responsibility of the teaching process is transferred to the students who have direct access to the content of the lessons before going to the physical classroom [6–9]. By providing students with online learning content in advance, flipped classroom learning increasingly engages learners. Blended learning has been extensively adopted and may become a new norm in higher education [10, 11]. Two recent meta-analyses have also provided reasonable evidence of its effectiveness in improving learners’ learning skills and their scores [12, 13].

The blended learning model contains six variations: flipped classroom, guided classroom time, integrated classroom time, capstone/independent learning, project-based and self-directed [14, 15]. And the flipped classroom model is the most common type of blended learning for undergraduate students. In addition to improving teaching quality, flipped classrooms are flexible enough to allow the implementation of various strategies in response to various course characteristics and the actual learning environment [6]. It can also be readily implemented alongside traditional learning methods in an existing course [16].

Various studies on blended learning in medical education have shown similar results. In anatomy learning, students displayed a positive attitude toward a blended learning method in radiologic anatomy [17]. The use of full-motion video from the anatomy dissection software really improved the experiences of the students in the dissection classes [18]. With a flipped and blended learning approach, first-year students have found histology more manageable than before [19], while social interactions have also improved without compromising practical skills [20].

Some limitations of blended learning have been noted in previous studies. Regardless of how well blended learning is designed, factors such as emotional and affective issues might hinder its stability and flexibility [21] and challenge the self-regulatory abilities of learners [22, 23]. While students are generally positive about a blended learning environment [24], a potential drawback of online near-peer anatomy teaching is that technological limitations and perceptions of online course instructors might lower student satisfaction [25].

In the blended learning of the histology practical, face-to-face learning is conducted in a virtual classroom. With the development of modern educational technology, virtual microscopy, slides, and virtual classrooms have already seen extensive and positive application in the histology practical, such that some educators have already converted all courses involving light microscopy into a virtual microscopy format [26–31] as the acquisition of histological knowledge is independent of the microscopy types of the learning material. According to a survey [29, 32] of histology learning from March 2020 to July 2020 during the COVID-19 pandemic in China, 50% of the responding schools (*n* = 39) showed a 50% increase in the use of virtual microscopy during online histology practical sessions; 48.7% (*n* = 38/78) implemented the online histology practical sessions in a flipped classroom (15%, *n* = 12) or via blended methods (33%, *n* = 26). The authors’ school was one of the schools that implemented online sessions incorporated blended learning for histology during the pandemic.

During the pandemic, switch of the learning histology practical from face-to-face to online worked smoothly, at least by appearance. However, we question the effectiveness of this switch. Even though the learning could be done without a physical microscopy and online learning is well-supported with virtual platform and software, we cannot help but wonder: is the flipped in virtual classroom of blended learning the same or better than physical classrooms? As a follow-up, would it be possible to permanently switch the learning format of histology practical into an online or a self-directed one in China? In this study, we compared the effectiveness of blended learning in histology practical between a flipped virtual classroom and a flipped physical classroom, examined the students’ knowledge acquisition and conducted a satisfaction survey in a blended learning experience of students within a flipped classroom model.

## Materials and methods

### Cohorts

Data were collected from three cohorts of sophomore medical students at the medical school of Zhejiang University (Hangzhou, China) during the regular histology practice in the summer terms of 2019, 2020, and 2021. There are 11 classes with different instructors for the histology practical each year, and the students will select a class before the summer semester. Each class consists of one instructor and 29–32 students who are either in a physical classroom (PCR) with face-to-face learning or a virtual classroom (VCR) with online learning supported by DingTalk (Alibaba, Hangzhou), a communication and collaboration platform.

There were 93 students across three flipped physical classrooms of blended learning (FPCR-BL) in 2021. There were 146 students across five flipped virtual classrooms of blended learning (FVCR-BL) in 2020, whereas there were 89 students across three physical classrooms employing traditional (didactic) learning (PCR-TL) in 2019.

### Histology practical course description

In the “organ and system-based learning” curriculum of the medical school of Zhejiang University, the learning units for MBBS sophomore students each summer semester are the cardiovascular system, immune system, and respiratory system. Students will learn 11 organ slides and some electrical-microscope images with instructors, such as the heart, blood vessels, lymph nodes, and lungs. For the e-contents, there were 10–15 min of mini-lectures from instructors for all slides recorded as videos, to which all students had open access. There were three sessions, each constituting approximately three learning hours.

### Knowledge acquisition assessment

Assessment comprised two quizzes for two sessions and a final exam for all sessions; all instructors participated in writing questions for quizzes and exams. Each quiz contained 20 single-choice questions to identify the required structure in an image, obtained from the histology question bank. The final exam contained 25 short answer questions, prepared by the department, to identify the required structure in an image and explain why.

### Histology practical homework

After reading the slides, students will complete their homework, which includes the following: 1) photographing the main structures and cells of all organs from classroom slides, labeling structures and cell names, and writing out their main characteristics; 2) describing the location and characteristics of its abnormal structure (excluding diagnosis) in a pathological slide belonging to the same system using the acquired histological knowledge; and 3) analyzing a series of changes in a group of post-intervention pictures copied from the research literature results of the same system using the acquired histological knowledge.

### Flipped classroom based on the blended learning model

**Fig. 1:**
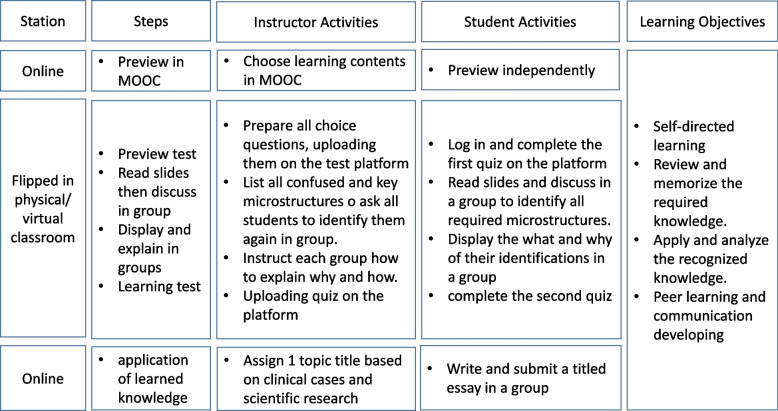
Blended Learning Design in Each Session of Histology Practical

A flipped classroom based on the blended learning model combines online and face-to-face learning collaboratively for the histology practical (Fig. 1) based on Bergman and Sam’s flipped classroom concept [30]. E-contents were released prior to the practical week. In the next scheduled face-to-face session, there was an interactive reading of glasses or virtual slides and a real-time discussion in groups of three to four students. The instructor discussed all points of confusion with the groups at any time and listened to the presentation of each group at the end of the session. Students in the virtual classroom communicated via DingTalk (Alibaba Group, Hangzhou, China).

### Data collection

All deidentified scores of the two quizzes and one final exam of the three groups were collected in September each year. An anonymous questionnaire about the effectiveness of blended learning in the FPCR-BL group was released on the “Questionnaire Star” platform in July 2021. This 11-item questionnaire with a five-point Likert scale used four factors to survey the effectiveness of blended learning: online learning satisfaction, classroom learning satisfaction, learning effects, and learning difficulty. The content validity of this questionnaire was confirmed by knowledgeable professors, and Cronbach’s alpha coefficient was used (80.0%) to assess its reliability.

The study was approved by the Institutional Review Board (IRB) committee of Zhejiang University (Approval No. 2019-jgyb20202001; 2020-zdjg21016). Informed consent was obtained from all participants included in the study.

### Statistical analysis

After the evaluation of normal distribution and analysis of variance, the scores were positively skewed, so the Kruskal–Wallis test method was used to compare the differences between groups. The questionnaire results were quantified and statistically analyzed. The Spearman method was used to analyze correlations between the scales of the four factors. The SPSS statistical software package for Windows (version 26.0; IBM Corp., Armonk, NY, USA) was used for data analysis.

## Results

### Participants

There were 89 students in the PCR-TL group, with an average age of 20.56 ± 2.21; 146 in the FVCR-BL group, with an average age of 20.20 ± 2.78; and 93 in the FPCR-BL group, with an average age of 20.61 ± 2.33. Chi-square statistical analysis showed that there was a significant difference in the percentage of male and female students (x^2^ = 7.014, *P* = 0.008) between FVCR-BL (male 39%; female 61%) and the other two classes (PCR-TL: male 58%; female 42%, FPCR-BL: male 51%; female 49%).

### Comparison of the quiz and final exam scores in three groups

There were significant statistical differences in all scores among the three groups based on nonparametric Kruskal–Wallis test (Table 1). The results showed that the quiz scores of the FVCR-BL and FPCR-BL groups were significantly higher than those of the PCR-TL group, and there was no statistical difference in quiz scores between the FVCR-BL and FPCR-BL groups. This suggests that the blended learning model improved the short-term memory of knowledge, exhibited by the increase in the scores of single-choice questions. In the final exam with short answer questions, the scores in FPCR-BL were higher than those in the other two groups, and there was no significant difference in the final exam between the PCR-TL and FVCR-BL groups. This means that the face-to-face element of the physical classroom in blended learning was helpful for a deeper understanding and application of knowledge.

**Table 1:** Comparison of histology practical quiz and exam scores in 3 groups

	PCR-TL Group (*n* = 89)	FVCR-BL Group (*n* = 146)	FPCR-BL Group (*n* = 93)	H	p
First Quiz Grade	80.00 (75.00–85.00)	95.00 (90.00–100.00)*	95.00 (90.00–100.00)*	106.28	0.00
Second Quiz Grade	80.00 (70.00–85.00)	95.00 (85.00–100.00)*	95.00 (85.00–100.00)*	75.43	0.00
Final Exam Grade	71.00 (53.00–80.00)	69.00 (54.00–77.00)	85.00 (73.00–95.00)^#^	69.68	0.00

*p<0.05: vs PCR-TL was significant; # *p*<0.05: vs FVCR-BL and FPCR-BL were significant. Data were expressed as median (IQR)

The effective size of the FPCR-BL group was slightly larger than that of the PCR-TL and FVCR-BL groups for classroom quizzes and final exam scores, with η_p_^2^ values of 0.386, 0.218, and 0.192, respectively, suggesting that the variation in scores of the FPCR-BL group was due to whether they engaged in the blended learning and in a flipped physical classroom.

### Questionnaire for flipped physical classroom with blended learning

A total of 90 questionnaires were collected from 93 students in the FPCR-BL group, which consisted of 37 males and 53 females; three students forfeited. The questionnaire had four factors and 11 items A five-point Likert scale was used in the investigation. Table 2 reports each of the 11 items that constitute the satisfaction scales of learning strategies and difficulties.

**Table 2:** Items and scores of blended learning questionnaire in FPCR-BL group (*n* = 90)

Factors	Items	Median	IQR
Online Learning	1. The MOOC preview is helpful for my study	5.00	4.00–5.00
	2. Preparatory quizzes are helpful to my study	5.00	4.00–5.00
	3. Group work is helpful for my study	4.00	3.00–5.00
Lab Learning	4. The teacher’s classroom guidance is suitable for my study	5.00	4.00–5.00
	5. Discussion among students in the group is helpful to my study	5.00	4.00–5.00
	6. It is helpful to show my classmates’ learning achievements	4.00	4.00–5.00
Learning Effect	7. Blended learning is helpful to my learning efficiency	4.00	4.00–4.00
	8. Blended learning is helpful to my learning method	4.00	4.00–4.00
Learning Difficulty	9. Quiz questions are relatively easy	2.00	2.00–3.00
	10. learning time of histology practice is less than others	3.00	3.00–4.00
	11. It is easier to read histology test slices	2.00	2.00–3.00

### Analysis of survey results in four factors

The questionnaire result options “strongly agree,” “agree,” “no option,” “disagree,” and “strongly disagree” were coded as “5,” “4,” “3,” “2,” and “1,” respectively. The results of each group were non-normally distributed according to the Kolmogorov–Smirnov test. The total Cronbach’s coefficient of the questionnaire was 0.800, and the validity KMO was 0.798. The coefficients of the 4 factors were as follows: online learning satisfaction (α = 0.730); classroom learning satisfaction (α = 0.751); learning effect (α = 0.611); learning difficulty (α = 0.596), which all reached the effective reliability.

Students’ perception scale of online learning was higher than that of classroom learning (4.03 ± 0.67). Students were satisfied with the blended learning effect (3.94 ± 0.54) and marked it a medium-level learning difficulty (3.05 ± 0.64). We also conducted correlation analyses to test the robustness of our findings on the relationships between the four factors of blended learning. Using Spearman correlation analysis, the results (Table 3) showed that there was a significant correlation between online learning and classroom learning (r = 0.581, *p* < 0.05), between online learning and learning effects (r = 0.378, *p* < 0.05), and between classroom learning and learning effects (r = 0.357, *p* < 0.05).

**Table 3:** Correlation analysis in 4 factors from questionnaire in FPCR-BL group (*n* = 90)

Items	Online learning	Classroom learning	Learning effect	Learning difficulty
Scores median(IQR)	4.67 (4.00–5.00)	4.00 (3.33–4.67)	4.00 (3.50–4.00)	3.00 (2.67–3.33)
Online Learning r	1	0.581*	0.378*	0.015
p		0.000	0.000	0.823
Classroom Learning r	0.581*	1	0.357*	0.084
p	0.000		0.000	0.223
Learning effect r	0.378*	0.357*	1	0.029
p	0.000	0.000		0.672
Learning difficulty r	0.015	0.084	0.029	1
p	0.823	0.223	0.672	

*The significance level was *p*<0.05

The results of the principal component analysis showed that the online learning and preview test of FPCR-BL design had the greatest impact on satisfaction; the percentage of variance was 51.51 and 16.80% respectively, with a cumulative percentage of 68.31%.

## Discussion

Histology practical have been supported by virtual classrooms for over 10 years in US [26], Germany [27], China [28] etc.. Learning in a virtual classroom, where students read scanned virtual slides using view software and not glass slides by microscope, provides more possibilities for the histology practical, especially during the COVID-19 pandemic. Compared to using a LM (light microscope) in traditional learning, some earlier studies have found that virtual slide reading was just as effective as glass slide reading for knowledge learning [29, 33]. Later studies [34] have also indicated that the virtual classroom was not only an effective method for teaching histology but also an effective assessment method for measuring student performance online, even during the COVID 19 pandemic period [35].

In our study, blended learning was more effective than traditional learning in a practical histology quiz. Compared with the traditional learning group (PCR-TL group), the quiz scores of the blended learning group (FVCR-BL group and FPCR-BL group) showed significant improvement. In contrast to traditional learning, blended learning requires a structural design to process extended learning step by step [7, 8]. This improvement correlates with the fact that in blended learning, students complete the online guidance videos for the classroom reading slides and feel confident for identifying the required structures of the quiz. Online learning materials are crucial for the results of the flipped classroom blended learning model, where instructors provide the resources to support flipping the classroom [36, 37]. In blended learning, students watched mini-lecture videos of each organ slide before class, but they did not do so in traditional learning. It is reasonable that the FPCR-BL and FVCR-BL groups had better quiz scores than the PCR-TL group, and that there was no difference between the FVCR-BL group and the FPCR-BL group.

Interestingly, the final exam scores showed contrary results to the two blended learning groups. The final exam scores of the FVCR-BL group were lower than those of the FPCR-BL group. An effective blended learning approach not only improves the short-term memorization of knowledge but also the integration between content and context by flipping. The FVCR-BL group was significantly different from the FPCR-BL group because they had a different flipped environment, namely, the physical/virtual classrooms. In a virtual classroom, the quantity and quality of the flipped interaction were reduced and the engagement of students was not as strong as that of the FPCR-BL group. Constructivism learning theory supports these results, stating that learners construct knowledge rather than simply passively taking in information, and that learning is inherently a social process because it is embedded within a social context given that students and teachers work together to build knowledge with affective [38–40]. Owing to the increased transactional distance in online environments, online interaction is often considered to be less spontaneous than face-to-face communication [41], which might cause feelings of learner isolation [42]. Students were generally dissatisfied with online learning, and they were especially dissatisfied with the communication and Q&A modes [43]. Online-only histology courses are effective for learning and are well accepted among students, whereas digital or remote learning has had positive effects during the COVID-19 pandemic [44]. However, there is a need for a synchronous learning environment with partial personnel-intensive small group settings to overcome passivity and inequality, and to foster active learning elements.

In the FPCR-BL group, physical classrooms with virtual slides or glass slides provided a real community for the knowledge building of histology. As outlined in constructivism theory, flipped physical classrooms are a crucial component of blended learning, even in the post-pandemic era. In addition to the interactive component, microscopy is another crucial learning tool for histology. Its value in medical training and medical practice has received some attention [45, 46]. In a survey of practitioners across a range of clinical settings on the use of their histology knowledge, it was found that 66% used microscopy weekly or daily, and approximately 90% of practitioners agreed that training in microscope use was essential in medical education [47]. A flipped virtual classroom using blended learning provides insufficient training and experience using a microscope to satisfy the needs of medical education.

The students in the FPCR-BL group had the highest satisfaction with online learning. Correlation analysis and principal component analysis in our study also showed that online learning was the main influencer of satisfaction. Online learning in our study had three activities provided by instructors and technicians. The online preview is from a course named “A Micro-world in Human Body - Histology Practical,” where each unit has several videos of reading slides and Q&As, that is published on the iCourse (China MOOC) platform, whereas the other two activities are self-directed quizzes and instantaneous feedback from the software that are both integrated into classroom learning. These findings indicate that higher levels of understandability, illustration, enthusiasm, and fostering the attention of online learning can lead to increased student satisfaction [48] and decreased frustration [49]. With the help of online activities, students can focus on solving difficulties and applying their knowledge in classroom learning; they also have more time to listen to their peers’ insights and engage in active conversations. Therefore, students should be provided with clear expectations, as well as numerous opportunities for self-paced and multimodal engagement with the target content both online and face-to-face [19, 50, 51].

The blended learning in histology practical supporting with virtual microscope and online resources has produced impressive improvements in learning efficiency and has notably enhanced the histology cognitive skills of students. Regardless, besides identification of histological structures, students should further develop critical thinking and analysis skills in medical issues [52, 53]. Importantly, a learner-centered blended learning, which combines online self-direct learning with face-to-face learning in the physical classroom will indeed increase the learning efficiency. It is time for histology staff members in medical schools toimprove the learning format and design of histology practical for MBBS students, especially in China.

## Limitations

The blended learning adopted in this study was self-designed and implemented based on the limited learning resources and instruments of Zhejiang University. Therefore, there is a lack of universal applicability for other institutions. Data from other classes in the academic years were not collected, and the non-flipped cohort did not have the benefit of discussing the slides. The learning effectiveness of blended learning in histology practical which evaluated with knowledge component was not fully convincing. Instructor teaching skills and student learning habits were also influential factors that were not examined. After calculation, the effective size of each group was relatively strong, but the evaluation rubric was not fully standardized. The effects of BL could be overestimated.

## Conclusions

Flipped classroom of blended learning includes two steps: the students’ asynchronous online learning and the instructor-led synchronous classroom learning, which are connected and integrated together. During the flipped classroom of blended learning in histology practical, the teaching that a traditional teaching offered was replaced by MOOC online lectures. Students preview the online resources following their schedule and process before a flipped learning in classroom. In our study, the quality of online resources directly affected student’s perception of blended learning, and it had close correlation with the satisfaction of classroom learning. The data analysis also showed that student’s perception of online learning and their pre-test scores was a majority component in all influence factors on the final scores. Although the histology practical learning has completed in online format in COVID-19 pandemic, this study found that online-only of blended learning had no satisfying learning effects and face-to-face in classroom was necessary for flipped classroom style of blended learning. In the post-pandemic era, online or blended learning in medical schools has received renewed attention; however, the classroom practical of medical courses still requires face-to-face instructions to help medical students overcome their academic and skill-related challenges.

## Data Availability

The datasets generated and/or analyzed in the current study are not publicly available due to participant informed consent formulated during the design of the study. Reasonable requests for dataset access can be granted by contacting the corresponding author.

## Abbreviations

FVCR-BL: Blended learning model flipped in a virtual classroom
FPCR-BL: Blended learning model flipped in a physical classroom
PCR-TL: Traditional learning model in a physical classroom
